# Effects of Recombinant Human Erythropoietin on Revascularization of Full Thickness Skin Grafts in Rat

**DOI:** 10.5812/ircmj.8867

**Published:** 2014-05-05

**Authors:** Mohammad Javad Fatemi, Abol Hasan Emami, Sina Ghiasi, Seyed Morteza Seyed Jafari, Ali Akbar Mohammadi

**Affiliations:** 1 Department of Plastic and Reconstructive Surgery, Fatemeh Zahra Hospital, Tehran University of Medical Sciences, Tehran, IR Iran; 2 Shiraz Burn Research Centre, Division of Plastic Surgery, Department of General Surgery, Shiraz University of Medical Sciences, Shiraz, IR Iran

**Keywords:** Erythropoietin, Neovascularization, Physiologic, Skin Transplantation

## Abstract

**Background:**

Autologous skin graft is frequently used in the field of plastic, and reconstructive surgery. The engraftment is dependent upon revascularization and angiogenesis, which can be regulated by different factors. In addition to its hematopoietic effects, erythropoietin is shown to positively affect the wound healing process.

**Objectives:**

We studied effects of human erythropoietin on revascularization of full thickness skin grafts in rat.

**Materials and Methods:**

Forty adult Albino male rats were selected for this study. Full thickness skin graft was performed for them, and the effects of systemic, and localized administration of erythropoietin on vascularization of the graft area were evaluated in four groups as following: inverse group underwent full thickness skin graft; in normal saline group normal saline was injected under the fascia of grafted area for seven days; systemic EPO group received systemic erythropoietin for seven days after the surgery; and in graft EPO group, erythropoietin was injected under the fascia of grafted area after full thickness skin grafting for seven days.

**Results:**

Forty adult Albino male rats (n = 40), with weights ranging from 356 to 469 g (mean 391.5 ± 29.6 g) were included. The vascular densities of central margins were significantly different between inverse group and graft EPO groups (P value = 0.01), and vascular density of central margins of normal saline group and graft EPO groups were significantly different too (P value = 0.04).

**Conclusions:**

EPO can stimulate angiogenesis which has an important role in wound healing. So, local administration of EPO seems to be beneficial in engraftment.

## 1. Background

Autologous skin graft is frequently used in the field of plastic, and reconstructive surgery (1), which is performed on the wounded area where the damaged skin has been removed (2). The engraftment take is dependent upon revascularization and angiogenesis occurs with sprouting and budding of vessels into the grafted tissue, from the recipient side (2, 3). Angiogenesis has an important role in the physiological wound-healing response which is mediated by cytokines and growth factors such as vascular endothelial growth factor (VEGF), fibroblast growth factor, and platelet-derived growth factor (4-6). New blood vessels serve as a pathway for oxygen and nutrient delivery, as well as for components of the inflammatory response (4). Erythropoietin (EPO) is a hematopoietic factor regulating the proliferation and differentiation of erythroid precursor cells (4, 7). Besides its hematopoietic effects EPO is shown to exert pleiotropic properties, such as cytoprotection, anti-inflammation, and antiapoptosis, and causes acceleration of wound epithelialization; it shortens the inflammatory phase, with faster resolution of the early granulation tissue (8).

The presence of EPO receptors on epithelial cells raised the hypothesis that EPO may directly affect the complex network of cytokines and growth factors involved in both the maturation of erythrocytes and the proliferation of endothelial cells (4, 9). EPO can stimulate the first initial phase of the angiogenic process (increase in cellular motility, cell matrix breakdown, and cellular proliferation) and the subsequent phase, which leads to the generation of new blood vessels from the pre-existing vessels (4, 5, 10).

## 2. Objectives

Because of all of the interesting characteristics of EPO, we studied effects of human erythropoietin on neovascularization of full thickness skin grafts in rat.

## 3. Materials and Methods

**Figure 1.**
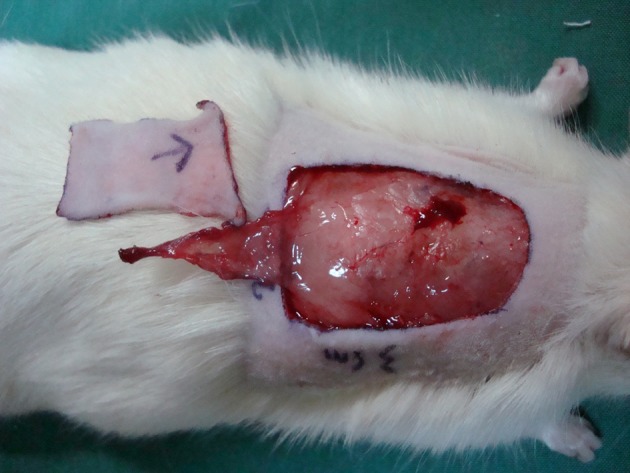
The Full Thickness Skin Graft Was Taken From the Area Between the Two Scapula Bones on the Dorsum of the Rat

**Figure 2.**
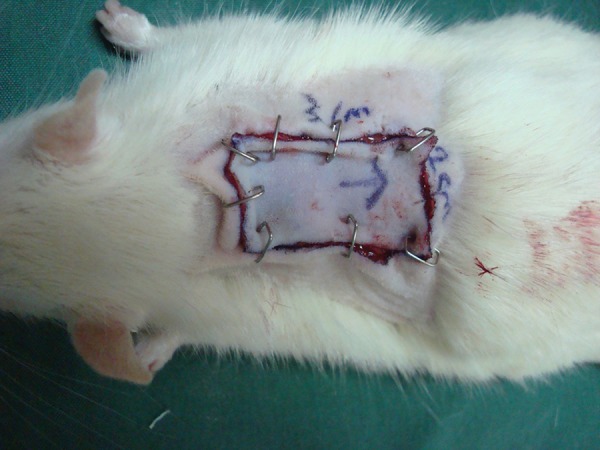
Skin Graft Was Performed in the Same Area From Which it Was Harvested After 180 Degrees Rotation in the Vertical Axis

Forty adult Albino male rats (n = 40), with weights ranging from 356 to 469 g (mean 391.5 ± 29.6 g), were selected for this study. During the experiments, animals were housed one per cage, maintained under controlled environmental conditions (12 hours light/dark cycle, temperature 23°C), and were provided with standard laboratory food and water ad libitum. The experiments were conducted in accordance with the Declaration of Helsinki and with the Guide for the care and use of laboratory animals. After induction of general anesthesia with ketamine hydrochloride (30 mg/kg intramuscularly) and xylazine (12 mg/kg intramuscularly), the surgical site was shaved and prepped with povidone iodine and chlorhexidine solution. Animals were covered with sterile surgical drapes with only the operative site exposed. A 2 × 3 cm full thickness skin graft was taken from the area between the two scapula bones on the dorsum of each rat (Figure 1). The skin graft was kept in normal saline before transferring to the recipient site. In this experimental model, each skin graft was repositioned in the same area from which it was harvested after 180 degrees rotation in the vertical axis (Figure 2).

The animals were randomly allocated into the following groups:

Group 1 (inverse group, n = 10): Underwent full thickness skin graft.
Group 2 (normal saline group, n = 10): After full thickness skin grafting, normal saline was injected under fascia of the grafted area for seven days.
Group 3 (systemic EPO group, n = 10): After performing surgery, the rats received systemic EPO (300 Unit/kg) for seven days.
Group 4 (Graft EPO Group, n = 10): After full thickness skin grafting, EPO (300 Unit/kg) was injected under the fascia of grafted area for seven days.

On the 7th day, rats were sacrificed and biopsies were taken from the grafted area. After fixation, staining of the samples with immunohistochemistry methods for CD31 and anti F-VШ, as accepted endothelial markers (4) was done. Then, vascular density (which is known as number of CD31+ and anti FVШ+ capillaries in each high power field) of different groups in anterior, posterior, and central margins of samples was reported by the pathologist.

### 3.1. Statistical Analysis

Data were collected, analyzed and reported as mean and standard deviation (mean ± SD). ANOVA test and post hoc comparison test were used for comparison of differences between four groups. Statistical analysis was carried out by using SPSS 19.0 software. P ≤ 0.05 was considered as statistically significant.

## 4. Results

Forty adult Albino male rats (n = 40), with weights ranging from 356 to 469 g (mean 391.5 ± 29.6 g) were included. ANOVA test showed significant difference in vascular density of central margins (P value = 0.005) (Table 1).

**Table 1.** Characteristics of Study Groups (n = 40) ^a^

	Total Number	Weight, g	Blood Vessels Count (Central Margin)	Blood Vessels Count (Anterior margin)	Blood Vessels Count (Posterior Margin)
**Inverse group**	10	402.0 ± 39.4	7.0 ± 3.3	9 ± 3.7	6.5 ± 1.9
**Normal saline group**	10	393.8 ± 33.5	7.6 ± 2.2	7.2 ± 2.6	5.5 ± 1.4
**Systemic EPO group**	10	384.6 ± 24.1	10.6 ± 3.2	7.4 ± 1.7	6 ± 1.3
**Graft EPO group**	10	385.6 ± 18.1	11.4 ± 3.4	9.6 ± 5.6	6.2 ± 2.6

^a^ Data are presented as Mean ± SD.

**Figure 3.**
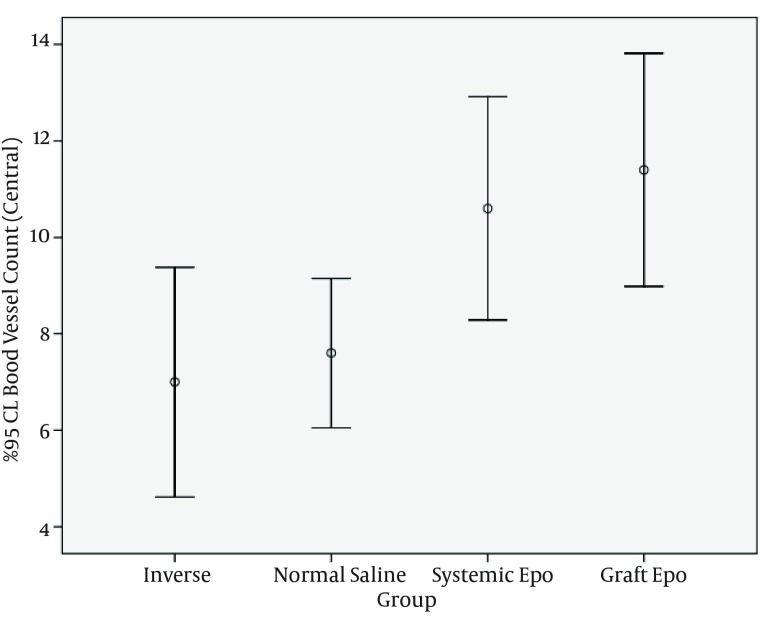
95 % CI Blood Vessels Count in Central Part of Different Study Groups

**Figure 4.**
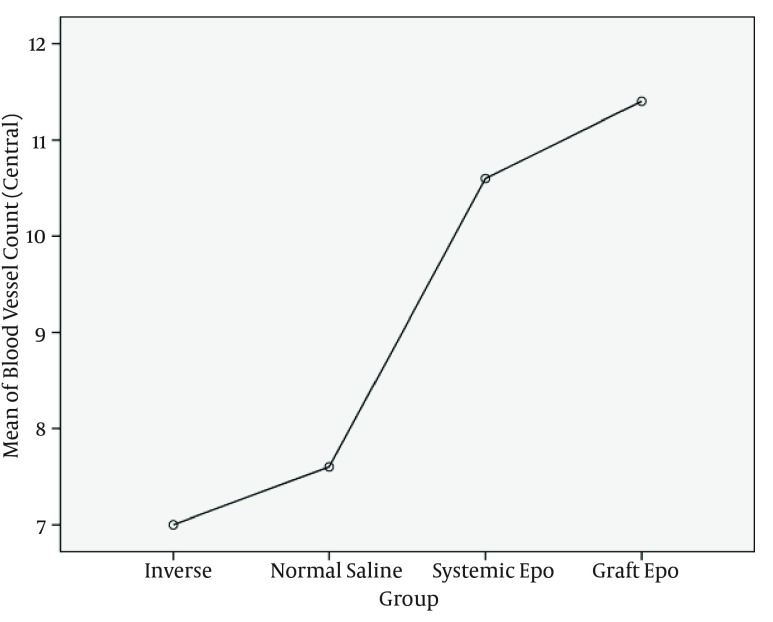
Mean Blood Vessels Count in Central Part of Different Study Groups

For analyzing the differences of central margins among the groups post hoc test was used. Vascular density of central margins was significantly different between inverse group and graft EPO group, (P value = 0.01), and vascular density of central margins of normal saline group and Graft EPO group showed statistical difference, too (P value = 0.04).

## 5. Discussion

The angiogenic cascade is known to result from a concerted interaction between the extracellular matrix, cells, and growth factors (4). After the initial angiogenic response, vessels from the recipient bed gradually take over the existing vascular structure of the graft with the newly formed vascular buds. Then endothelial cells initially migrate into the perivascular space creating gradually enlarging vascular lumens which in turn are covered by migrating pericytes from the inner side of the vascular wall (3). Besides hematopoietic capability, EPO stimulates mitosis and induces differentiation of numerous cell lines, such as the endothelial, myocardial, smooth muscle, and mesangial cells, through acting as a growth factor (4, 10).

Enhancement of neovascularization by EPO has been attributed to direct effect of EPO on VEGF production and on endothelial cell mitosis (8, 11). The novel physiological role of EPO is a pro-angiogenic role (5) i.e. enhancing angiogenesis in granulation tissues suggesting that the pro-healing effect of EPO is associated with its stimulatory effect on the proliferation and migration of mature endothelial cells during wound healing (5, 8, 12). The angiogenic effect of EPO was seen in our study, where local administration of EPO significantly increased the vascular density in grafted skins. EPO is further found to stimulate the proliferation and tube formation of cultured neonatal micro vascular endothelial cells (8, 13). In addition, EPO is shown to enhance inflammation- and ischemia-induced neovascularization (9). EPO can stimulate angiogenesis which has an important role in wound healing. So, local administration of EPO seems to be useful for engraftment. Obviously, clinical application of our results requires further evaluations in human subjects.
